# Supplementary material for: Anticancer Activity and Molecular Targets of Piper cernuum Substances in Oral Squamous Cell Carcinoma Models
Source: Biomedicines. 2023 Jul 6;11(7):1914. doi: 10.3390/biomedicines11071914 (PMC10377665; doi:10.3390/biomedicines11071914)
Supplement: Supplementary file 1 [file biomedicines-11-01914-s001.zip › biomedicines-2477062-supplementary.pdf]

Anticancer Activity and Molecular Targets of *Piper cernuum* Substances in Oral Squamous Cell Carcinoma Models

Thaíssa Queiróz Machado[1,†], Maria Emanuelle Damazio Lima[2,†], Rafael Carriello da Silva[3], Arthur Ladeira Macedo[4], Lucas Nicolau de Queiroz[1], Bianca Roberta Peres Angrisani[2], Anna Carolina Carvalho da Fonseca[3], Priscilla Rodrigues Câmara[5], Vitor Von-Held Rabelo[6], Carlos Alexandre Carollo[4], Davyson de Lima Moreira[7], Elan Cardozo Paes de Almeida[5], Thatyana Rocha Alves Vasconcelos[2], Paula Alvarez Abreu[6], Alessandra Leda Valverde[2,*], Bruno Kaufmann Robbs[5,*]

[1] Postgraduate Program in Applied Science for Health Products, Faculty of Pharmacy, Fluminense Federal University, Niteroi, RJ, Brazil.

[2] Department of Organic Chemistry, Chemistry Institute, Fluminense Federal University, Niteroi, RJ, Brazil.

[3] Postgraduate Program in Dentistry, Health Institute of Nova Friburgo, Fluminense Federal University, Nova Friburgo, RJ, Brazil.

[4] Pharmaceutical Sciences, Food and Nutrition Faculty, Mato Grosso do Sul Federal University, Campo Grande, MS, Brazil.

[5] Basic Science Department, Health Institute of Nova Friburgo, Fluminense Federal University, Nova Friburgo, RJ, Brazil.

[6] Biodiversity and Sustainability Institute, Macaé Campus, Federal University of Rio de Janeiro, Macaé, RJ, Brazil.

[7] Research Directorate, Laboratory of Natural Products and Biochemistry, Rio de Janeiro Botanical Garden Research Institute, Rio de Janeiro, RJ, Brazil.

SUPPLEMENTARY METHODS

Prediction of the mechanism of action of the main phytochemicals of the studied fractions

To predict the mechanism of action of the compounds responsible for the biological activity, we used different molecular modeling strategies based on the five main phytochemicals found in the fraction. Compounds were separated into two groups according to their chemical similarity. Boldine and coclaurine were selected as representative compounds for each group. First, a similarity search was conducted within PDB and ChEMBL databases to select potential targets for these compounds. Second, a pharmacophore search was performed using the Pharm Mapper webserver [1]. The search was conducted using the human proteins-only target set which contains

2,241 pharmacophore models for human proteins. For protein selection, a normalized fit score of 0.9 was used as the cutoff and compounds must have fulfilled all the pharmacophore criteria. Finally, for both approaches, proteins were chosen according to experimental evidence in the literature that correlates them with anticancer effects, particularly in OSCC.

After target selection, inverse docking studies were carried out between the five compounds of the fractions and all proteins. Protonation states (pH 7.4) of the compounds were predicted using the MarvinSketch 16.2.29 program, 2016, ChemAxon (http://www.chemaxon.com). *R* and *S* enantiomers of coclaurine and *N*-methylcoclaurine were considered in our study. 3D structures of the compounds were constructed using RDKit and further submitted to geometry optimization using OpenBabel 3.1 (http://openbabel.org/). For optimization, structures were subjected to 2,500 cycles of energy minimization using the steepest descent method, followed by a conformational analysis with the weighted method and 2,500 cycles of energy minimization using the conjugate gradient method. The MMFF94 force field was applied in all steps. Finally, partial charges were calculated using the semi-empirical method EEM based onDFT-B3LYP/6-311G/NPA. Proteins were prepared in Autodock Tools 1.5.7 or Hermes 2022.3 by removing solvent molecules, and other artifacts and adding hydrogens. For docking studies with CK2, the conserved water molecules W1 (588) and W2 (510) were considered while the conserved water molecule W (808) was considered for Dyrk1A studies. Different docking protocols and programs were used and they are summarized in **Supplementary Table 1**. In all simulations, the search space was centered on the ligand. Other parameters that are not mentioned were kept as default. All docking protocols were validated by redocking studies and RMSD values of the top-scoring poses were lower than 2 Å (**Supplementary Table 1**). The top-scoring pose of each ligand with each protein was selected for scoring normalization and interaction analysis using Pymol 2.5 (The PyMOL Molecular Graphics System, Version 2.5, Schrödinger, LLC) and Discovery Studio Visualizer 2021 (Dassault Systèmes BIOVIA, San Diego, 2021). Docking scores obtained from Autodock 4.2.6 were negated and, then, the values were normalized by using the combined Z-score method [2] for comparison among the different targets.

SUPPLEMENTARY DATA

Supplementary Table S1: Docking parameters and validation results used in the inverse docking studies for different proteins. RMSD was calculated after the superimposition of the experimental pose and the top-scoring binding pose predicted in the molecular docking study.

Protein	PDB code	Docking program	Docking protocol	Search algorithm	RMSD (Å)
AR	2PIP	Gold 2022.3	Search radius = 10 Å;<br>GA runs = 30;<br>Scoring function = ASP;<br>Search efficiency = 70%.	Genetic algorithm	0.84
Chk1	2CGW	Gold 2022.3	Search radius = 10 Å;<br>GA runs = 30;<br>Scoring function = ChemPLP.	Genetic algorithm	1.60
CK2	6HNY	Gold 2022.3	Search radius = 10 Å;<br>GA runs = 30;<br>Scoring function = ChemPLP.	Genetic algorithm	0.44
Dyrk1A	4YLK	Autodock 4.2.6	Grid box: 40x40x40 (0.375 Å spacing);<br>GA runs = 50.	Lamarckian genetic algorithm	0.53
EHMT2	7X73	Gold 2022.3	Search radius = 11 Å;<br>GA runs = 30;<br>Scoring function = Goldscore;<br>Search efficiency = 200%.	Genetic algorithm	0.70
LXRβ	1PQ9	Autodock 4.2.6	Grid box: 40x40x40 (0.375 Å spacing);<br>GA runs = 30;<br>Max Number Evaluations = 1,500,000.	Lamarckian genetic algorithm	1.06
VEGFR2	3CJF	Gold 2022.3	Search radius = 11 Å;<br>GA runs = 30;<br>Scoring function = Goldscore and rescore with ASP;<br>Search efficiency = 200%.	Genetic algorithm	1.78

Supplementary Table S2: Table of the average per group of the histopathological findings on chronic toxicity study for dichloromethane fraction of the leaves of *Piper cernuum*. The results were common to the treated and control animals. There was hyperemia in the lungs, liver and kidneys, which was similar in mice of all groups and may be associated with euthanasia stress. Legend: +: discrete / ++: moderate / +++: accentuated; N: No; Y: Yes; N/C: No changes.

Organ	Histopathological findings	Control			PCLd			4NQO			4NQO + PCLd		
		PBS + DMSO			480mg/kg			100µg/ml			100µg/ml + 480mg/kg		
Lung	Alveolar edema	N	N	N	N	N	N	N	N	N	N	N	N
	Arterial and venous hyperemia	+	++	+	++	+	++	+++	++	+++	+++	++	+
	Compensatory emphysema	+	+	+	+	+	+	+	+	+	+	+	+
	Obvious regional lymph node	Y	N	N	Y	N	N	N	N	Y	N	Y	N
Heart	Myocardium	N/C	N/C	N/C	N/C	N/C	N/C	N/C	N/C	N/C	N/C	N/C	N/C
	Valves	N/C	N/C	N/C	N/C	N/C	N/C	N/C	N/C	N/C	N/C	N/C	N/C
	Base Vessels	N/C	N/C	N/C	N/C	N/C	N/C	N/C	N/C	N/C	N/C	N/C	N/C
Kidney	Hyperemia	++	+	+	+	+	+	+	+	+	+	+	+
Liver	Centrolobular hyperemia	N	N	N	+	N	N	N	N	N	N	N	+
	Portal hyperemia	++	++	++	++	++	++	++	++	++	+	++	++
	Megalocytosis	+	+	+	+	+	+	+	+	+	+	+	+
	Intracytoplasmic vacuolar degeneration	+	+	+	+	+	+	+	+	+	+	+	+
	Obvious hepatocyte cords	N	N	N	N	N	N	N	N	N	N	N	N
	Perivascular and periportal lymphocyte focus	Y			Y			Y			Y		
	Hepatocyte Binucleation	1 focus	N	Y	3 focus	N	N	1 focus	N	Y	1 focus	N	N
Spleem	Red Pulp	1 focus	N		+	+	+	1 focus			+	+	+
	White Pulp	+	+	+	N/C	N/C	N/C	+	+	+	N/C	N/C	N/C

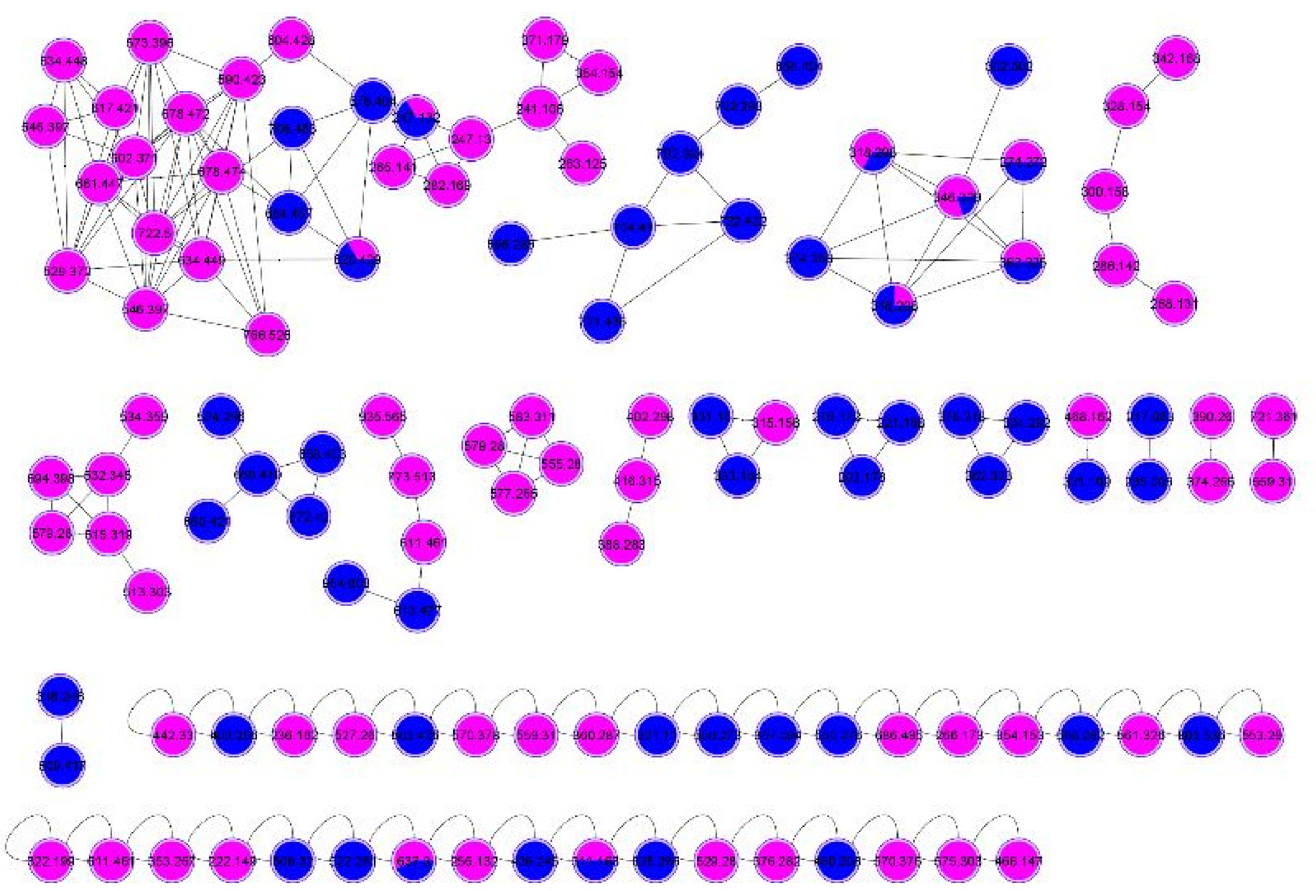

Supplementary Figure S1: Molecular Network of Fractions 09.07 and 14.05.

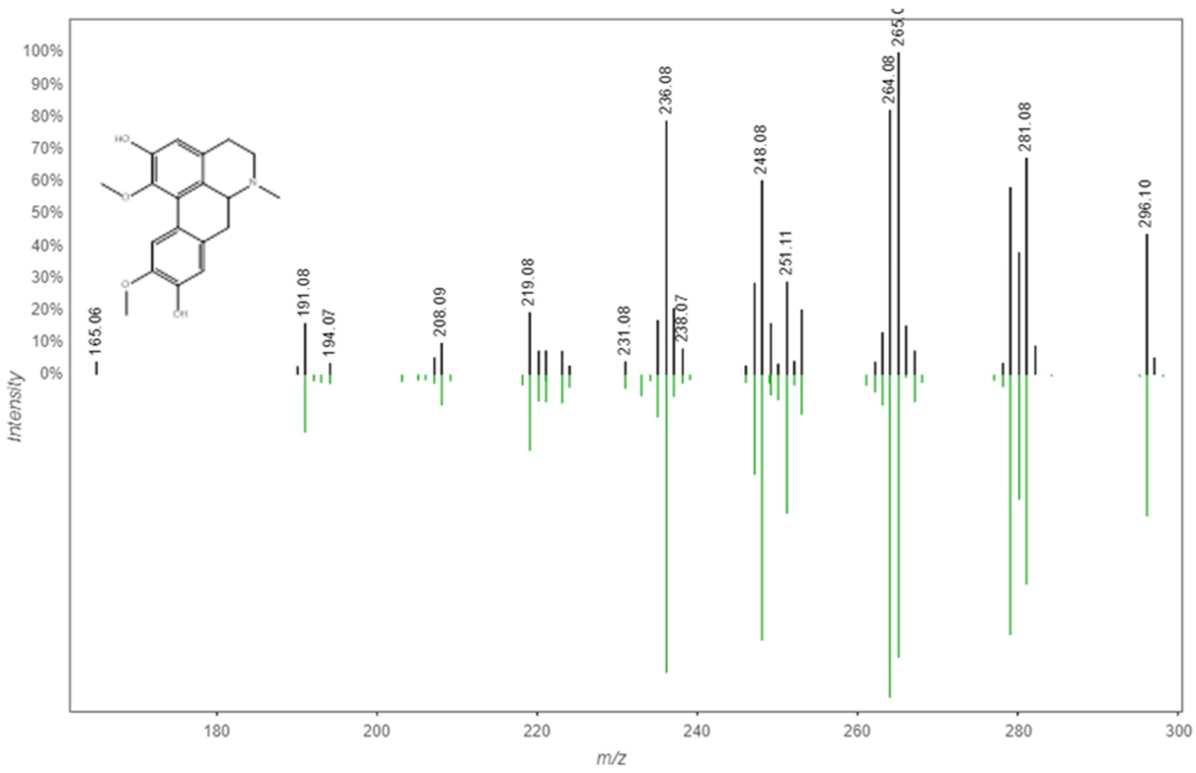

Supplementary Figure S2: Comparison of the obtained spectrum and the isocorydine library (**2**).

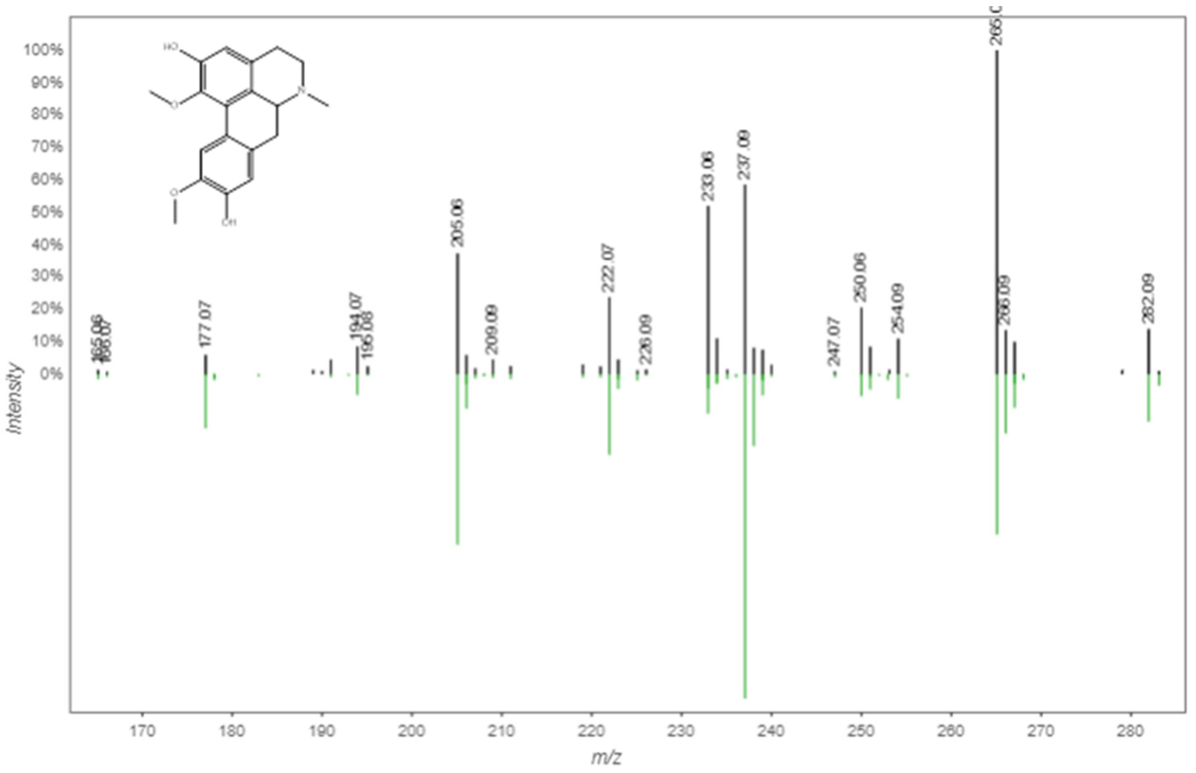

Supplementary Figure S3: Comparison of the obtained spectrum and the boldine library (**3**).

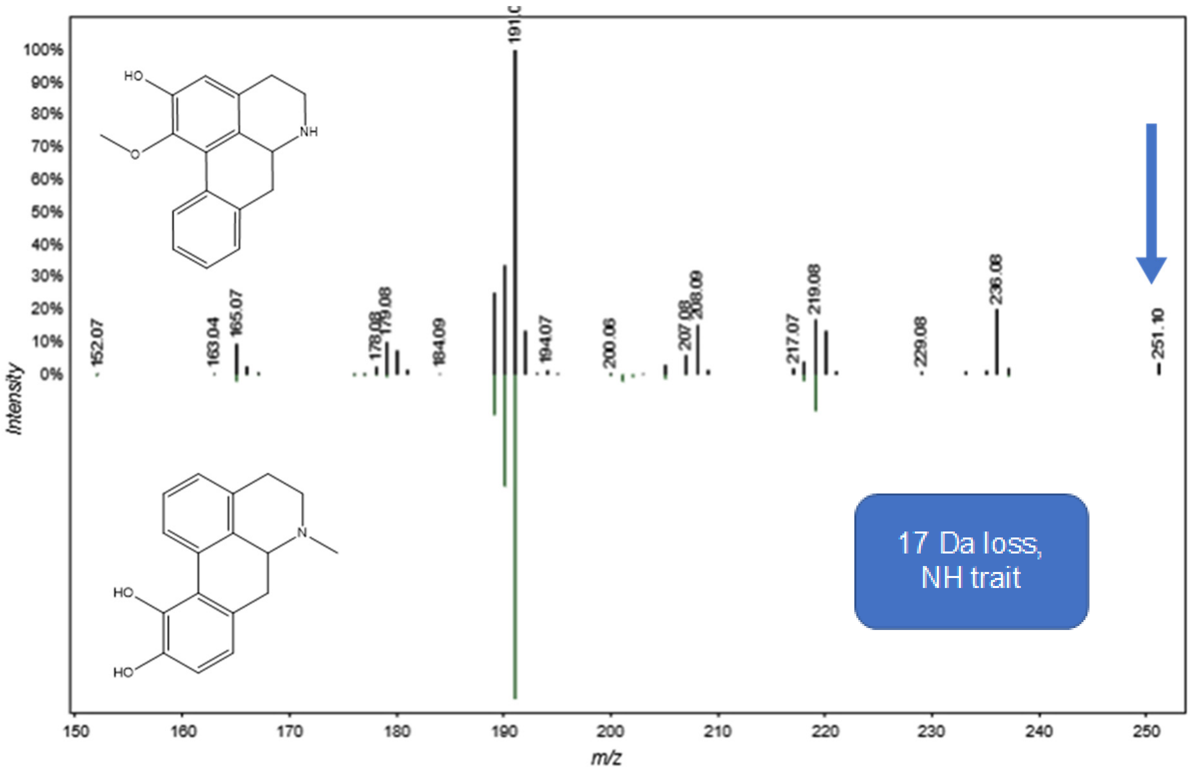

Supplementary Figure S4: Comparison of the spectrum obtained from asimilobine (**3**).

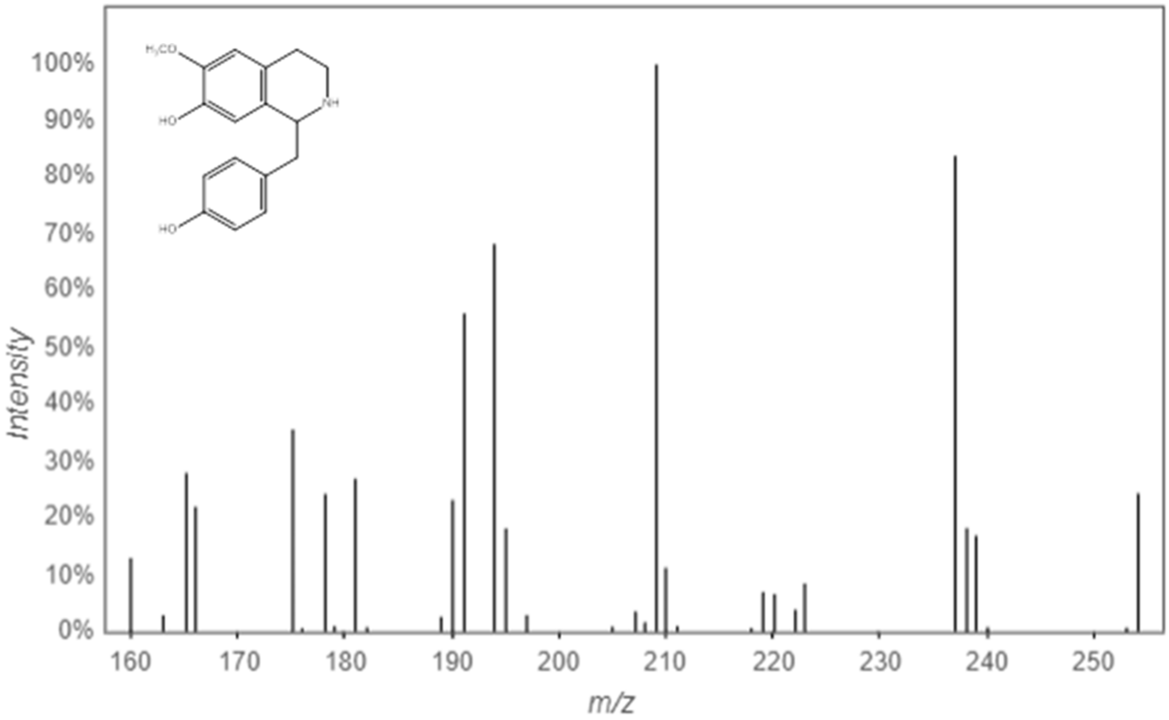

Supplementary Figure S5: Spectrum obtained from coclaurine (**4**).

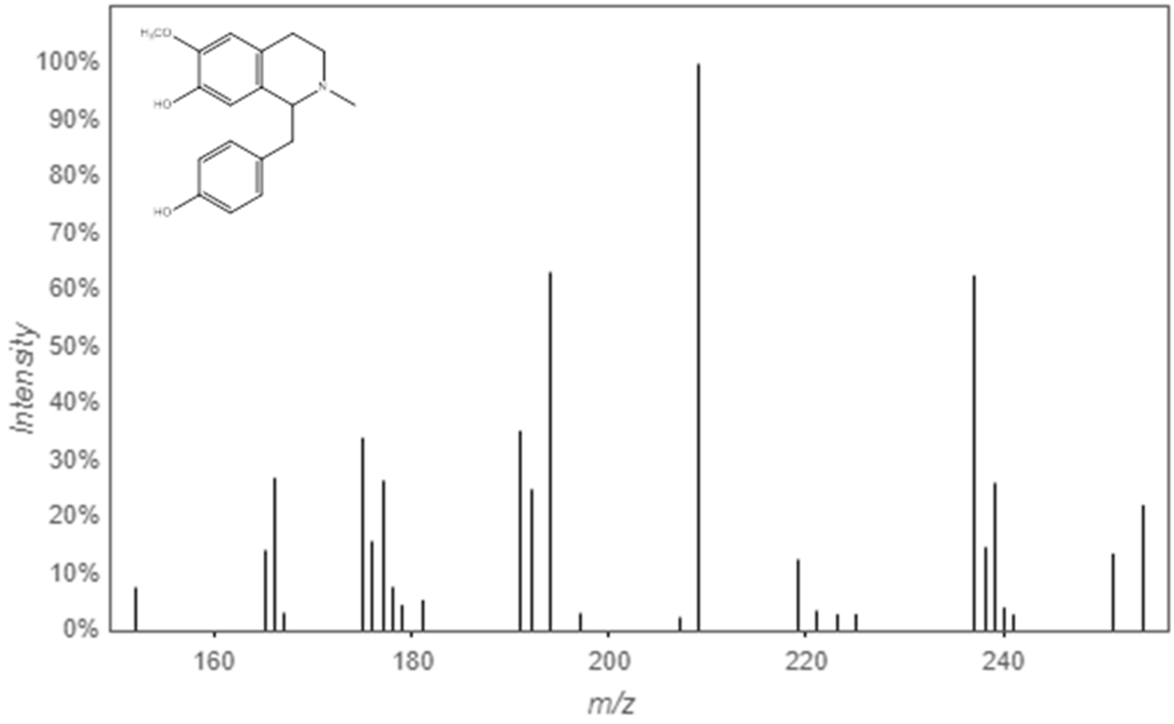

Supplementary Figure S6: Spectrum obtained from *N*-methylcoclaurin (**5**).

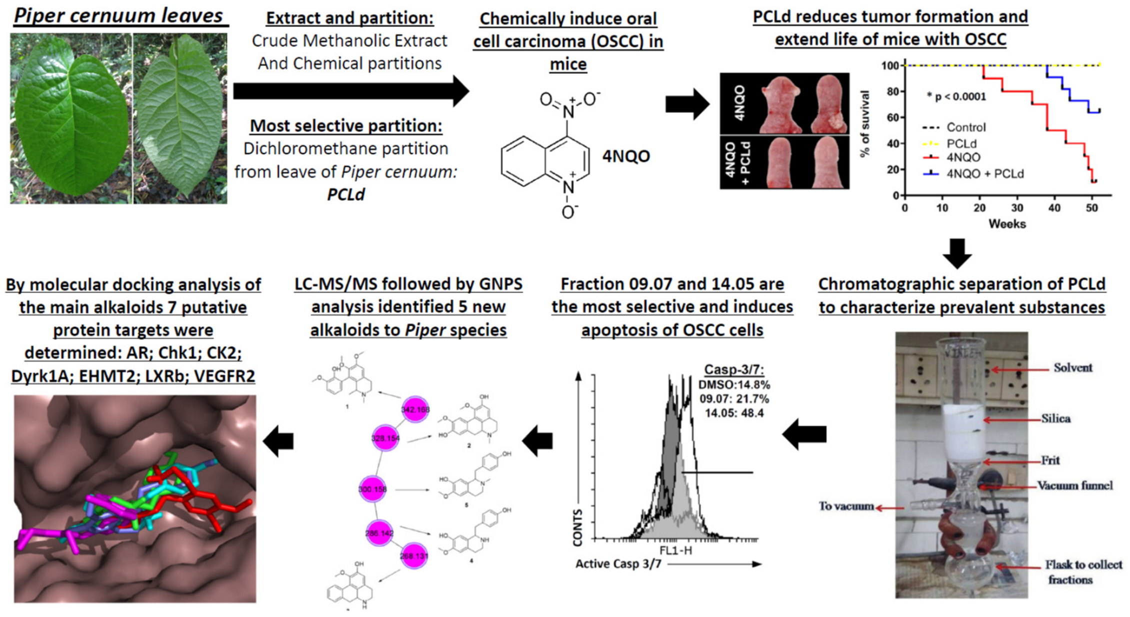

Supplementary Figure S7: Scheme demonstrating the main findings of the work.

References:

1. Wang, M.; Carver, J.J.; Phelan, V.V.; Sanchez, L.M.; Garg, N.; Peng, Y.; Nguyen, D.D.; Watrous, J.; Kapono, C.A.; Luzzatto-Knaan, T.; et al. Sharing and community curation of mass spectrometry data with Global Natural Products Social Molecular Networking. *Nat. Biotechnol.* **2016**, *34*, 828–837.

2. Kim, S.S.; Aprahamian, M.L.; Lindert, S. Improving inverse docking target identification with Z-score selection. *Chem. Biol. Drug Des.* **2019**, *93*, 1105–1116.
